# Internal Disulfide Bonding and Glycosylation of Interleukin-7 Protect Against Proteolytic Inactivation by Neutrophil Metalloproteinases and Serine Proteases

**DOI:** 10.3389/fimmu.2021.701739

**Published:** 2021-06-30

**Authors:** Jennifer Vandooren, Rafaela Vaz Sousa Pereira, Estefania Ugarte-Berzal, Vasily Rybakin, Sam Noppen, Melissa R. Stas, Eline Bernaerts, Eva Ganseman, Mieke Metzemaekers, Dominique Schols, Paul Proost, Ghislain Opdenakker

**Affiliations:** ^1^ Laboratory of Immunobiology, Rega Institute for Medical Research/KU Leuven, Department of Microbiology, Immunology and Transplantation, Leuven, Belgium; ^2^ Laboratory of Virology and Chemotherapy, Rega Institute for Medical Research/KU Leuven, Department of Microbiology, Immunology and Transplantation, Leuven, Belgium; ^3^ Laboratory of Molecular Immunology, Rega Institute for Medical Research/KU Leuven, Department of Microbiology, Immunology and Transplantation, Leuven, Belgium

**Keywords:** IL-7, matrix metalloproteinase-9, proteolysis, signal transduction, proliferation, neutrophils

## Abstract

Interleukin 7 (IL-7) is a cell growth factor with a central role in normal T cell development, survival and differentiation. The lack of IL-7–IL-7 receptor(R)-mediated signaling compromises lymphoid development, whereas increased signaling activity contributes to the development of chronic inflammation, cancer and autoimmunity. Gain-of-function alterations of the IL-7R and the signaling through Janus kinases (JAKs) and signal transducers and activators of transcription (STATs) are enriched in T cell acute lymphoblastic leukemia (T-ALL) and autocrine production of IL-7 by T-ALL cells is involved in the phenotypes of leukemic initiation and oncogenic spreading. Several IL-7-associated pathologies are also characterized by increased presence of matrix metalloproteinase-9 (MMP-9), due to neutrophil degranulation and its regulated production by other cell types. Since proteases secreted by neutrophils are known to modulate the activity of many cytokines, we investigated the interactions between IL-7, MMP-9 and several other neutrophil-derived proteases. We demonstrated that MMP-9 efficiently cleaved human IL-7 in the exposed loop between the α-helices C and D and that this process is delayed by IL-7 N-linked glycosylation. Functionally, the proteolytic cleavage of IL-7 did not influence IL-7Rα binding and internalization nor the direct pro-proliferative effects of IL-7 on a T-ALL cell line (HPB-ALL) or in primary CD8^+^ human peripheral blood mononuclear cells. A comparable effect was observed for the neutrophil serine proteases neutrophil elastase, proteinase 3 and combinations of neutrophil proteases. Hence, glycosylation and disulfide bonding as two posttranslational modifications influence IL-7 bioavailability in the human species: glycosylation protects against proteolysis, whereas internal cysteine bridging under physiological redox state keeps the IL-7 conformations as active proteoforms. Finally, we showed that mouse IL-7 does not contain the protease-sensitive loop and, consequently, was not cleaved by MMP-9. With the latter finding we discovered differences in IL-7 biology between the human and mouse species.

## Introduction

Interleukin (IL)-7 plays multiple non-overlapping roles throughout normal T cell development, homeostasis, and activation. In the thymus, IL-7 produced by thymic epithelial cells drives survival and proliferation of developing thymocytes and regulates the timing of their differentiation (1–3). In the periphery, IL-7 is an important pro-survival factor for naïve T cells and is required for their homeostatic proliferation (4, 5). Memory CD8^+^ T cells also rely on IL-7 for survival (6). These effects of IL-7 are mediated by several downstream pro-survival factors, such as Bcl-2 and Mcl-1 (7). In lymph nodes, a delicate balance between IL-7 production, mainly by stromal cells, and its consumption by several subsets of innate lymphoid cells orchestrates T cell proliferation, as was recently demonstrated (8).

IL-7 signals through a heterodimeric receptor composed of a cytokine-specific α-subunit (CD127) and a common γ-chain (γ_c_/CD132) which is shared with other IL-2 family cytokine receptor complexes (9). Binding of IL-7 results in the formation of a signaling-competent receptor complex, cross-phosphorylation of Jak1 and Jak3 bound to IL-7Rα and the γ_c_-chain, respectively, and phosphorylation of IL-7Rα, which then acts as a signaling platform for Stat5 and other signaling molecules (9, 10). In CD8^+^ T cells, the ectodomain of IL-7Rα is shed from the cell surface upon the interaction with IL-7, reaching its lowest levels 24-48 h after stimulation (11).

Human regulatory T cells (Treg) do not express IL-7Rα (12). The ability of IL-7 to promote proliferation of naïve and memory T cells, but not Treg, is in stark contrast with the effects of IL-2 (13–15). In patients with idiopathic CD4 lymphopenia, injections of recombinant human IL-7 promoted bursts of proliferation of CD4^+^ and CD8^+^ T cells. An increase in CD4^+^ and CD8^+^ T cells was also observed in a study employing patients with chronic HIV-1 infection, where application of IL-7 increased proliferation of both peripheral T cells and thymocytes, as seen by an increase in recent thymic emigrants (16, 17). Aberrations in the IL-7/IL-7R/JAK/STAT signaling pathway have also been associated with leukemia, with particular bad prognosis in adult patients with T-ALL (18–20). Therefore, studies of the behavior, bioavailability and functional effects of IL-7 are relevant in biology and medicine.

We recently showed that IL-2, another γ_c_ family cytokine, is efficiently degraded by neutrophil-derived proteases, both in biochemical and in cell-based assays (21). Whereas neutrophil elastase (NE), proteinase 3 (P3) and cathepsin G (catG) degraded IL-2 completely, matrix metalloproteinase (MMP)-9 produced a family of stable products which retained the ability to bind the IL-2 receptor and to initiate signaling, albeit with a timing and magnitude clearly different from those of the intact cytokine. An investigation of the cleavage of other γ_c_ family cytokines by MMP-9 identified IL-7 and IL-21 as targets, while IL-4, IL-9, and IL-15 were barely or not cleaved (21). These findings added more targets to the growing repertoire of chemokines and cytokines processed by MMP-9. Reports from our and other laboratories have clearly indicated that MMP-9-mediated cleavage regulates the biological activity of such molecules. MMP-9 strongly potentiates IL-1β and IL-8, whereas cleavage of IL-2 and the chemokine CCL2 reduces their potency in target cell assays (22, 23). Therefore, the activity of MMP-9, which is known to be significantly elevated at sites of acute inflammation, due to the degranulation of neutrophils and other cell types (24, 25), has the potential to affect cellular responses to soluble signaling factors. In addition, MMP-9 cleaves the ectodomains of several critically important cell surface molecules, such as the IL-2 receptor α-subunit CD25 (26).

In the present study, we investigated the proteolytic processing of IL-7 by neutrophil proteases. Specifically, we identified new cleavage sites in the sequence of IL-7 for the proteases MMP-9, NE and P3. Furthermore, we showed that posttranslational modifications, specifically internal disulfide bonding and glycosylation, protect IL-7 against proteolysis by neutrophil proteases and against functional inactivation and that the latter proteolysis is species-specific.

## Materials and Methods

### Proteins, Reagents, and Buffers

The following preparations of recombinant human IL-7 were obtained; human IL-7 produced in *E. coli* (Peprotech, cat. no. 200-07), human IL-7 produced in insect cells (Novus, cat. no. NBP2-52629) and human IL-7 produced in human HEK-293 cells (Biolegend, cat. no. 581904) (see **Supplementary Table 1**). The IL-7 receptor ectodomain was purchased from R&D Systems (rhIL-7, cat. no. 306-IR-050). Recombinant human MMP-9 was produced, purified, activated and tested as described (27). Recombinant human proMMP-2, proMMP-7, proMMP-8 and ‘a disintegrin and metalloproteases’ (ADAM)17 were purchased from R&D Systems and activated as previously described (28). Active human NE was purchased from Abcam (cat. no. ab91099).

### Proteolytic Processing of IL-7 and His-Tag Pull-Down

Recombinant human proteases were added to IL-7 at a molar ratio of 1/100 (protease/IL-7), unless indicated otherwise, in a buffer containing 150 mM NaCl, 10 mM CaCl_2_, 30 mM Tris and 0.01% Tween-20 (pH 7.4). Reactions were incubated at 37°C for 4 hours, unless mentioned otherwise. Histidine tag pull-down was performed with the use of Ni-NTA magnetic microbeads, according to the manufacturer’s instructions (ThermoFisher, cat. no. 88832).

### SDS-PAGE, Western Blot, and Edman Sequencing

IL-7 samples were separated in Novex 16% tricine gels (Invitrogen, cat. no. EC6695BOX) as recommended by the supplier. Proteins in gels were stained with the SilverQuest Silver Staining Kit (Invitrogen cat. no. LC6070) or transferred onto PVDF membranes using the Trans-Blot Turbo Transfer System with associated materials and protocols (Biorad, cat. no. 1704150). For Edman sequencing, proteins on the PVDF membrane were stained with Coomassie Brilliant Blue (Coomassie R-250, Thermo Scientific, cat. no. 20278) and analyzed with the Procise 491cLC protein sequencer (Applied Biosystems) or the PPSQ-51A protein sequencer (Shimadzu). For the analysis of signaling molecules, proteins in cell lysates were separated in 4-12% Tris-glycine gels under reducing conditions and transferred to PVDF membranes. Membranes were blocked for 1 h in 5% BSA with TBST buffer (150 mM NaCl, 0.1% Tween 20, 50 mM Tris, pH 7.5) and incubated overnight with anti-pSTAT3 (Tyr^705^) (Cell Signaling, cat. no. 9138) or anti-β-actin (Proteintech, cat. no. 20536-1-AP) antibodies. After washing, the blot was incubated with peroxidase-conjugated anti-mouse IgG or anti-rabbit IgG for 1 h at room temperature. Finally, Western blot images were developed using the Vilber Lourmat Fusion system (Labtech International) and Pierce ECL Western Blotting Substrate (Thermo Fisher Scientific).

### Binding Assays

Surface Plasmon Resonance (SPR) experiments were carried out on a Biacore T200 instrument (GE Healthcare). The recombinant subunits of the IL-7 receptor were immobilized onto the surface of carboxymethylated dextran chips (CM5, GE Healthcare). Experiments were performed at 25°C in 10 mM HEPES, 150 mM NaCl, 3 mM EDTA and 0.05% Tween 20. Several concentrations of intact IL-7 or cIL-7 (IL-7 cleaved by MMP-9) were injected for 2 min at a flow rate of 30 μl/min, after which a 5 min dissociation phase was maintained. The sensor chip surface was regenerated with 4 M MgCl_2_. Binding affinities (K_D_) and kinetic rate constants (k_on_ and k_off_) were derived after fitting the experimental data to the two-state reaction model (IL-7-IL-7Rα) in the Biacore T200 Evaluation Software 2.0 and according to McElroy et al. (29).

### Cell Culture and Proliferation Experiments

HPB-ALL cells (human T cell leukemia cell line) were purchased from the German cell culture collection (DSMZ, cat. no. #ACC 483). Cells were maintained in RPMI-1640 supplemented with 10% fetal calf serum (FCS). For stimulation, indicated amounts of intact recombinant human IL-7 or cIL-7 were added to the cells and cell growth was monitored by using the Incucyte live-cell analysis system and associated software (Essen BioScience). For flow cytometry analysis of the IL-7R, 0.5x10^6^ HPB-ALL cells were stimulated with 50 ng/ml IL-7 or left untreated for the indicated period of time.

### Flow Cytometry

Cells were washed with ice-cold PBS and incubated for 15 min with the Fc-receptor-blocking antibodies anti-CD16/anti-CD32 (BD Biosciences Pharmingen). After washing with PBS + 2% FCS, cells were stained for 20 min. HPB-ALL cells were stained with the phycoerythrin (PE)-conjugated anti-CD127/IL7Rα (Biolegend, cat. no. 351342) and human peripheral blood mononuclear cells (PBMCs) were stained with phycoerythrin (PE)-conjugated anti-CD8 (Biolegend, cat. No. 344706), allophycocyanin (APC)-conjugated anti-CD127/IL7Rα (Biolegend, cat. no. 351315) and a Zombie Aqua™ viability dye (BioLegend). Cells were washed twice, fixed with 0.37% formaldehyde and analyzed on a BD LSR Fortessa X20 with DIVA software (BD Biosciences, v9.0). Results were further analyzed with the FlowJo software package (BD Biosciences, v10.0). An example of the gating strategies is illustrated in **Supplementary Figure 4**.

### Isolation and Stimulation of Human PBMCs

PBMCs were isolated from 1-day old buffy coats derived from blood donated by healthy volunteers (Blood Transfusion Center of the Belgian Red Cross, Mechelen, Belgium) with the use of Lymphoprep (Axis-Shield). Cells were suspended in RPMI-1640 medium supplemented with 10% FCS, 1 mM sodium pyruvate, and 1% GlutaMAX at a packing of 10^6^ cells/ml and stimulated with 500 pM of recombinant human IL-7 or cIL-7. The cells were further analyzed by flow cytometry.

### Human Neutrophil Degranulates and Incubations

Neutrophil degranulates were collected from human blood granulocytes stimulated with N-formylmethionyl-leucyl-fenylalanine (fMLF), as previously described (21, 28). All blood donors gave written informed consent in accordance with the Declaration of Helsinki. Neutrophil degranulates from different donors were pooled, to minimize individual-based variation. To evaluate IL-7 digestion by neutrophil proteases, 1 µg IL-7 was incubated with 5 µl of neutrophil degranulate (37°C, 6 hours), in the presence or absence of a metalloprotease inhibitor (100 mM EDTA), serine protease inhibitor (1mg/ml Pefabloc), aspartic protease inhibitor (0.7 ug/ml pepstatin) or cysteine protease inhibitor (10 µg/ml E-64).

## Results

### MMP-9 Efficiently Cleaves Human IL-7 and This Proteolysis Is Influenced by the IL-7 Glycosylation State

**Figure 1:**
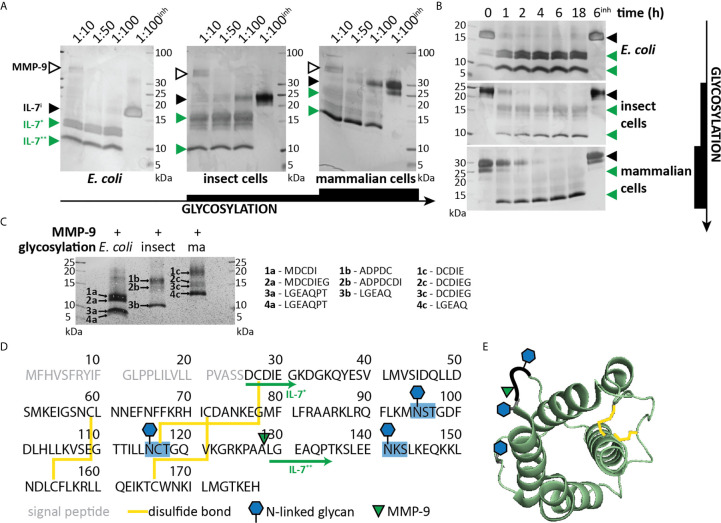
Characterization of IL-7 cleavage by MMP-9. **(A)** Recombinant IL-7 produced in *E. coli* (left), insect cells (middle) or human HEK-293 cells (right) were incubated with different concentrations of active MMP-9 at the indicated molar ratios (MMP-9/IL-7), for a period of 2h. Fragments were separated by SDS-PAGE under reducing conditions followed by silver staining. MMP-9, intact IL-7 (IL-7^i^) and two cleavage products (IL-7* & IL-7**) are indicated with arrowheads, respectively, in black and green color. ^inh^; negative control in the presence of 500 µM of the MMP inhibitor SB-3CT (inh). **(B)** Recombinant IL-7 produced in *E. coli* (top, non-glycosylated), insect cells (middle, partially glycosylated) or human HEK-293 cells (bottom, fully glycosylated) were incubated with active MMP-9 at a molar ratio of 1:100 (MMP-9/IL-7) and samples were taken at the indicated time-points. Fragments were resolved by SDS-PAGE under reducing conditions. Intact IL-7 (IL-7^i^) and two cleavage products (IL-7* & IL-7**) are indicated with arrowheads, respectively, in black and green color. ^inh^; negative control in the presence of 500 µM of the MMP inhibitor SB-3CT. **(C)**, Edman sequencing data. Identification of the neo-N-termini generated upon digestion of IL-7 with MMP-9 (1:100, MMP-9:IL-7 for 6h). Identification of the LGEAQ N-terminus from fragment IL-7**, indicates an MMP-9 cleavage site between the amino acid residues A^128^ and L^129^. Variations in N-terminal amino acids of fragments IL-7* are due to the different IL-7 expression systems (**Supplementary Table 1**). **(D)** Amino acid sequence of human IL-7 with indication of glycosylation sites (blue), disulfide bridges (yellow), MMP-9 cleavage site (green triangle) and IL-7 fragments (green arrows). **(E)** 3D model of IL-7 [PDB structure 3DI3 (29)] with indication of the location of the MMP-9 cleavage site (green triangle), glycosylation sites (blue hexagons) and disulfide bridges (yellow).

We previously investigated the *in vitro* cleavage of IL-2 family cytokines by MMP-9 and identified IL-7 as an MMP-9 substrate (21). Given the importance of IL-7 in T cell proliferation, we aimed at further characterizing this interaction. Since glycosylation is known to influence the interactions between IL-7 and IL-7Rα (29), we performed our experiments with non-glycosylated IL-7 (produced in *E. coli*), with glycosylated IL-7 produced in insect cells and with glycosylated human IL-7 manufactured in human HEK-293 cells (**Supplementary Table 1**). Incubation of IL-7 with active human MMP-9 generated at least two prominent IL-7 fragments (IL-7* and IL-7**, **Figures 1A, B** and **Supplementary Figure 1**). Non-glycosylated IL-7 was most efficiently cleaved with complete cleavage as soon as 1 hour after incubation at a molar ratio of 1:100 (MMP-9/IL-7). In contrast, glycosylated IL-7 was less efficiently cleaved, requiring higher enzyme:substrate ratios (**Figure 1A**) and longer incubation periods (**Figure 1B**) for full digestion. N-terminal sequencing analysis resulted in the identification of the neo-N-terminal fragment LGEAQ, representative for a cleavage between amino acid residues Ala^128^ and Leu^129^ (**Figures 1C, D**). This cleavage site was also confirmed by *in silico* digestion of IL-7 with MMP-9, using the iProt-Sub package (30) and was present in the exposed loop between α-helices C and D (29, 31) **(** **Figure 1E**).

### IL-7 Fragments Derived From MMP-9 Proteolysis Remain Connected Through Disulfide Bridges and Remain Functionally Active

**Figure 2:**
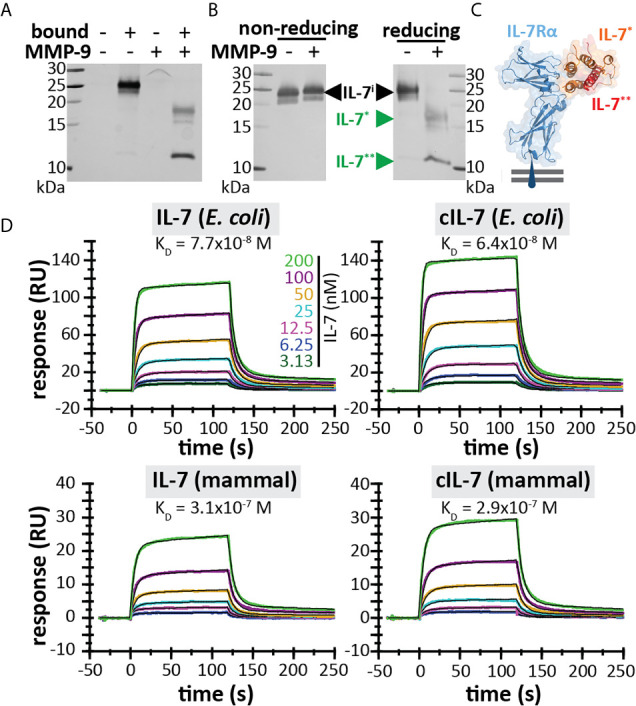
IL-7 interaction with the IL-7 receptor remains intact upon cleavage by MMP-9. **(A)** His-tagged IL-7 (produced in insect cells) was subjected to histidine tag pull-down. Bead-bound proteins and non-bound proteins were resolved by reducing SDS-PAGE. **(B)** IL-7 in the presence (+) or absence (-) of MMP-9, subjected to non-reducing or reducing electrophoretic separation. **(C)** 3D model of the interaction between IL-7 and the IL-7Rα (blue) [based on PDB structure 3DI3 (29)] with indication of the IL-7 fragments generated by MMP-9 [IL-7* (orange) and IL-7** (red)]. **(D)** Binding of IL-7 [from *E. coli* (top) or mammalian cells (bottom)] and MMP-9-cleaved IL-7 (cIL-7, 1/100 molar ratio MMP-9/IL-7, 4h at 37°C) to rhIL-7Rα, as analyzed by SPR (results representative for 2 experiments, see **Supplementary Figure 3**). Colors represent different concentrations of IL-7 as indicated and black lines represent curve fits with a two-state reaction model (IL-7-IL-7Rα) according to McElroy et al. (29).

**Figure 3:**
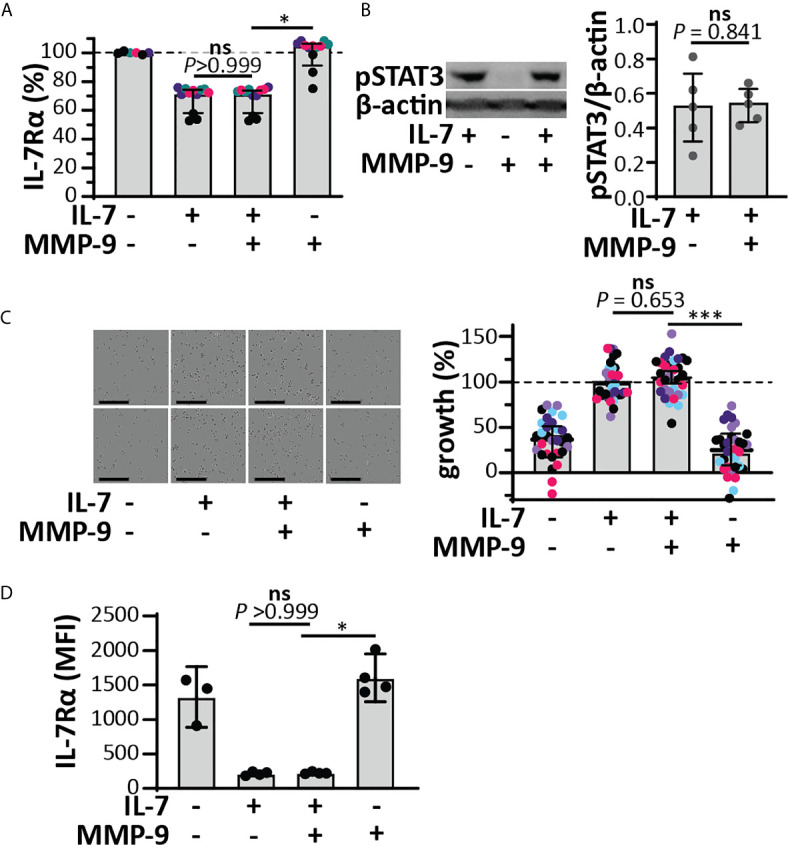
Functional effects of IL-7 and IL-7-receptor internalization in HPB-ALL cells and human CD8^+^ PBMCs remain intact upon cleavage by MMP-9. **(A)** Analysis of cell-surface IL-7R, 5 minutes after stimulation of HPB-ALL cells with 50 ng/ml IL-7, cIL-7 (1/100 molar ratio MMP-9/IL-7, 4h at 37°C) or the equivalent of MMP-9. Data represent four independent experiments, with experimental replicates shown in the same color. Histograms represent median values and error bars represent the IQR. Data were normalized to the unstimulated condition, representing steady-state quantities of cell surface IL-7R (100% IL-7Rα). *p < 0.05, as determined by Kruskal-Wallis test with Dunn’s correction for multiple comparisons and calculated on mean values of each experiment. **(B)** Analysis of pSTAT3 (Tyr^705^) in HPB-ALL cells, 15 minutes after stimulation with 50 ng/ml IL-7, cIL-7 (1/100 molar ratio MMP-9/IL-7, 4h at 37°C) or the equivalent of MMP-9. Each data point indicates an independent experiment. Histograms represent median values and error bars represent the IQR. Data were normalized to β-actin. **(C)** Growth of HBP-ALL cells, 4 days upon stimulation with 50 ng/ml IL-7, cIL-7 (1/100 molar ratio MMP-9/IL-7, 4h at 37°C) or the equivalent of MMP-9 (right panel). Data represent six independent experiments, with all experimental replicates shown in the same color. Histograms represent medians and error bars represent the IQR. Data were normalized to cells stimulated with IL-7 alone, representing 100% growth. ***p ≤ 0.001, as determined by Kruskal-Wallis test with Dunn’s correction for multiple comparisons and calculated on the mean values of each experiment. (Left panel) representative images of HPB-ALL cells with the indicated stimulations. Scale bar = 200 µm. **(D)** Analysis of cell-surface IL-7R, 24h after stimulation of human CD8^+^ PBMCs with 15 ng/ml IL-7, cIL-7 (1/100 molar ratio MMP-9/IL-7, 4h at 37°C) or the equivalent of MMP-9. Histograms represent median values and error bars represent the IQR. *p < 0.05, as determined by Kruskal-Wallis test with Dunn’s correction for multiple comparisons. ns, not significant.

Since IL-7 is able to form three internal disulfide bridges (**Figures 1D, E**), we wondered whether under native conditions and after cleavage by MMP-9, the IL-7 fragments would remain connected or function as separate protein fragments. His-tagged IL-7 (at the C-terminus) was digested with MMP-9 and the C-terminal fragment was pulled down by nickel bead precipitation (**Figure 2A**). Even when digested by MMP-9, the N-terminal fragment of IL-7 precipitated together with the C-terminal fragment, indicating that the fragments remain attached under native conditions. Furthermore, under denaturing but non-reducing conditions (electrophoresis with SDS and without reducing agent) the fragments also remained together, indicating a strong interaction, based on disulfide bridge formation (**Figure 2B**). These results were confirmed for glycosylated variants, produced by insect and mammalian cells (see **Supplementary Figure 2**). Next, we wondered whether the cleavage by MMP-9 would influence IL-7 function. IL-7 binds to a heterodimeric complex consisting of the IL-7Rα and the common γ-chain [shared with the receptors for other interleukins (9)] and induces survival and proliferation of thymocytes (29, 32). To mimic the interaction between IL-7 and IL-7Rα, we immobilized the IL-7Rα ectodomain on SPR chips and analyzed the binding of IL-7 and cIL-7 (**Figures 2C, D**). Interestingly, modification of IL-7 by MMP-9 proteolysis did not reduce its ability to bind to the IL-7Rα chain. The dissociation constants of intact IL-7 *versus* cleaved IL-7 (cIL-7) were always in the same order of magnitude for aglycosyl (*i.e.* without glycosylation) *E. coli*-derived (7.7 x 10^-8^ *versus* 6.4 x 10^-8^), and mammalian cell glycoproteins (3.1 x 10^-7^ *versus* 2.9 x 10^-7^). Therefore, the binding to the IL-7Rα chain was always similar for the intact and cleaved IL-7. It was also possible to compare the effect of glycosylation on the affinities towards the IL-7Rα chain. We found that the presence of N-linked oligosaccharides decreased the affinities for IL-7Rα on the IL-7 protein and these effects were observed for both the intact and cleaved IL-7 glycoforms. (**Figure 2D** and **Supplementary Figure 3**). Upon binding to the IL-7Rα, IL-7 induces rapid clathrin-mediated internalization of the IL-7/IL7R complex, which is required for activation of downstream signal transduction (33). To investigate this mechanism, we used the human T cell leukemia cell line HPB-ALL. Addition of IL-7 to the growth medium resulted in a rapid decrease of the IL-7Rα at the cell surface (**Supplementary Figures 4** and **5A**), induced IL-7-mediated signal transduction (**Supplementary Figure 5B**) and stimulated growth of HPB-ALL cells under serum-free conditions (**Supplementary Figure 5C**). Interestingly, cleavage of IL-7 by MMP-9 did not alter the ability of IL-7 to induce receptor internalization (**Figure 3A**), to activate pSTAT3 (**Figure 3B** and **Supplementary Figure 6**) or to induce HPB-ALL proliferation (**Figure 3C**). Finally, both IL-7 and cIL-7 could reduce IL-7R levels at the surface of CD8^+^ human PBMCs with equal efficiency, 24 hours after IL-7 treatment (**Figure 3D**). At 24h after IL-7 stimulations, CD8^+^ PBMCs are known to also induce IL-7R shedding, a known late functional effect of IL-7 (11).

### Human IL-7 Is Efficiently Cleaved by Other Neutrophil-Derived Metalloproteinases and Serine Proteases

**Figure 4:**
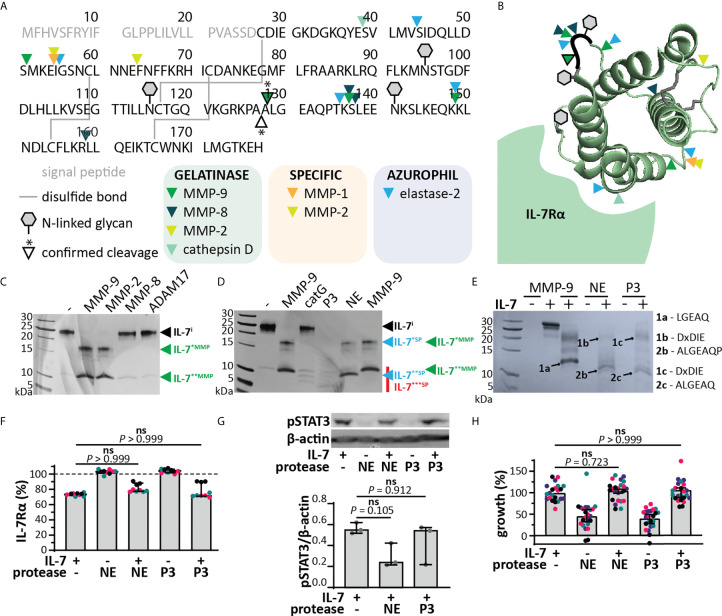
IL-7 signaling through the IL-7 receptor remains intact upon cleavage by neutrophil proteases. **(A)** *In silico* prediction of IL-7 proteolysis by neutrophil proteases (iProt-Sub tool). Separation of proteases based on their place in neutrophil granules including gelatinase (green), specific (orange) and azurophil granules (blue). **(B)** 3D model of IL-7 [PDB structure 3DI3 (29)] with indication of the location of the predicted cleavage sites for neutrophil proteases. **(C)** Digestion of IL-7 with a selection of metalloproteinases. Digestions at a molar ratio of 1/100 (MMP/IL-7) and for 4h. IL-7^i^, intact IL-7; fragments are indicated by asterisks (*). **(D)** Digestion of IL-7 with the serine proteases catG, P3 and NE. Digestions at a molar ratio of 1/100 (protease/IL-7) and for 4h. **(E)** Edman sequencing data. Identification of the neo-N-termini generated upon digestion of IL-7 with NE or P3 (1:100, protease:IL-7 for 4h). Identification of the ALGEAQ N-terminus from fragment IL-7**^SP^, indicates a shared cleavage site between the amino acid residues A^127^ and A^128^. **(F)** Analysis of cell-surface IL-7R, 5 minutes after stimulation of HPB-ALL cells with 50 ng/ml IL-7, cIL-7 (1/100 molar ratio MMP-9/IL-7, 4h at 37°C) or the equivalent of the indicated protease. Data represent three independent experiments, with all experimental replicates shown in the same color. Histograms represent median values and error bars represent the IQR. Data were normalized to the unstimulated condition, representing steady-state quantities of cell surface IL-7R (100% IL-7Rα). Statistical analysis done by Kruskal-Wallis test with Dunn’s correction for multiple comparisons and calculated on mean values of each experiment. **(G)** Analysis of pSTAT3 (Tyr^705^) in HPB-ALL cells, 15 minutes after stimulation with 50 ng/ml IL-7, cIL-7 (1/100 molar ratio protease/IL-7, 4h at 37°C). Each data point indicates an independent experiment. Histograms represent median values and error bars represent the IQR. Data were normalized to β-actin. **(H)** Growth of HBP-ALL cells, 4 days upon stimulation with 50 ng/ml IL-7, cIL-7 (1/100 molar ratio protease/IL-7, 4h at 37°C) or the equivalent of MMP-9. Data represent four independent experiments, with all experimental replicates shown in the same color. Histograms represent medians and error bars represent the IQR. Data were normalized to cells stimulated with IL-7 alone, representing 100% growth. Statistical analysis done by Kruskal-Wallis test with Dunn’s correction for multiple comparisons and calculated on mean values of each experiment. ns, not significant.

MMP-9 is mainly associated with myeloid cells, in particular it is found in secretory granules of neutrophils and released at sites of acute and excessive inflammation, together with other metalloproteinases (*e.g.* MMP-8, ADAM-17) and serine proteinases (*e.g.* NE, catG and P3) (34, 35). *In silico* analysis revealed that several other neutrophil granule-held proteases (34) could potentially cleave IL-7 (**Figures 4A, B**). We performed *in vitro* cleavage experiments with a selection of metalloproteinases and serine proteases characteristic to neutrophils. All tested metalloproteinases were able to cleave IL-7, generating similar fragments as MMP-9 (IL-7*^MMP^ and IL-7**^MMP^, **Figure 4C**). Cleavage by MMP-8 and ADAM17 was less efficient than by MMP-9, whereas cleavage by MMP-2 (known for its high similarity with MMP-9) was similar to that by MMP-9. The serine proteases catG, P3 and NE were also able to cleave IL-7 (**Figure 4D**). Proteolysis of IL-7 by catG was least efficient, however, proteolysis by P3 and NE resulted in one or more smaller fragments (IL-7***^SP^). NE generated two fragments, similar to MMP-9, although with lower molecular weight (IL-7**^SP^). N-terminal sequencing of IL-7^**^SP^^ and IL-7^***^SP^^ resulted in the identification of the neo-N-terminal fragment ALGEAQ, representative for a cleavage between amino acid residues Ala^127^ and Ala^128^ (**Figure 4E**), differing only one amino acid from the MMP-9 cleavage site. This cleavage site was not predicted *in silico*.

### IL-7 Proteolysis by Action of Neutrophil Proteases Does Not Alter IL-7-Mediated Proliferation in a T Cell Leukemia Cell Line

**Figure 5:**
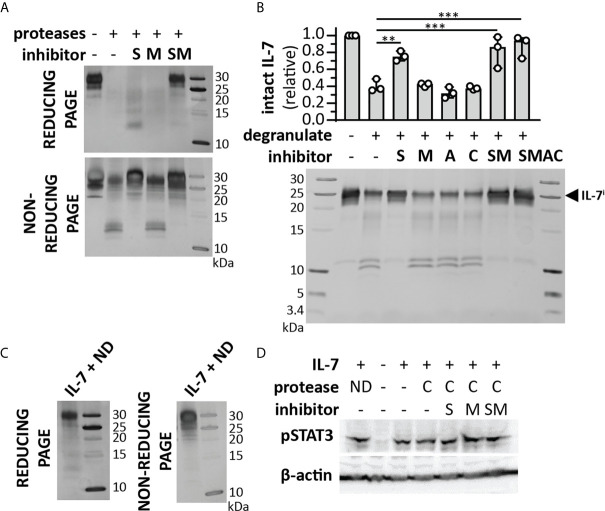
Cooperative cleavage of IL-7 by neutrophil proteases generates fragments that remain interconnected and functionally intact. **(A)** Treatment of IL-7 with a cocktail of neutrophil proteases (MMP-9, P3 and NE, at a molar ratio of 1/100 (protease/IL-7) and for 4h) in the presence or absence of a serine protease inhibitor (S), metalloproteinase inhibitor (M) or their combinations (SM). Analysis of the proteins by reducing SDS-PAGE illustrates the disappearance of intact IL-7 and diffuse staining at lower molecular weights. In contrast, under non-reducing conditions, the majority of the IL-7 signal re-appears. **(B)** Cleavage of IL-7 by proteases present in neutrophil degranulates (5 µl from a pool of 5-10 donors and incubation with 1µg IL-7) in the presence or absence or a serine protease inhibitor (S), metalloproteinase inhibitor (M), aspartic acid protease inhibitor (A), cysteine protease inhibitor (C) or their combinations. Top panel, densitometry analysis of intact IL-7 (IL-7^i^). Data were normalized to intact IL-7 in the condition without neutrophil degranulate. **p ≤ 0.01, ***p ≤ 0.001, as determined by Kruskal-Wallis test with Dunn’s correction for multiple comparisons (n = 3). Bottom panel, representative image of SDS-PAGE analysis. **(C)** IL-7 digested with neutrophil degranulate (ND) and run under non-reducing conditions, results in the re-appearance of IL-7 at higher molecular weight. **(D)** Induction of pSTAT3 by IL-7, upon treatment with a cocktail (C) of neutrophil proteases (MMP-9, P3 and NE, at a molar ratio of 1/100 (MMP/IL-7) and for 4h) in the presence or absence of a serine protease inhibitor (S), metalloproteinase inhibitor (M) or their combinations (SM) or with neutrophil degranulate neutrophil degranulate (ND). β-actin is shown as a loading control.

Since the cleavage of IL-7 by serine proteases resulted in different fragments, we also evaluated the effect of this proteolysis on IL-7 functionality. Interestingly, cleavage of IL-7 by NE or P3 did not alter IL-7R internalization (**Figure 4F**). Although a reduction in pSTAT3 signaling was observed for NE (**Figure 4G** and **Supplementary Figure 7**), this effect was not significant, nor did it result in a significant effect on proliferation of the T cell line HPB-ALL (**Figure 4H**). Next, we investigated the combined action of neutrophil proteases MMP-9, NE and P3. Treatment of IL-7 with this protease cocktail resulted in the disappearance of intact IL-7 on reducing SDS-PAGE analysis (**Figure 5A**). Interestingly, under non-reducing conditions, the signal of higher molecular weight IL-7 re-appeared, except for one lower molecular weight form generated by serine proteases. Furthermore, incubation of neutrophil degranulates from human donors with IL-7 resulted in the disappearance of intact IL-7 and appearance of IL-7 fragments similar to IL-7*^MMP^, IL-7*^SP^, IL-7**^MMP^ and IL-7**^SP^ (**Figure 5B**). Only the serine protease inhibitor Pefabloc was able to inhibit the formation of IL-7 fragments, indicating that neutrophil serine proteases are the main proteases cleaving IL-7 in neutrophil degranulates. In addition, under non-reducing conditions, the loss in signal of intact IL-7 was restored, again pointing towards the fact that IL-7 fragments remain interconnected after proteolysis (**Figure 5C**). Finally, we also investigated whether IL-7 would remain functionally intact. Indeed, IL-7 treated with a neutrophil protease cocktail or neutrophil degranulates, remained capable of inducing STAT3 phosphorylation (**Figure 5D**).

### Mouse IL-7 Does Not Contain the Protease Sensitive Loop Region

**Figure 6:**
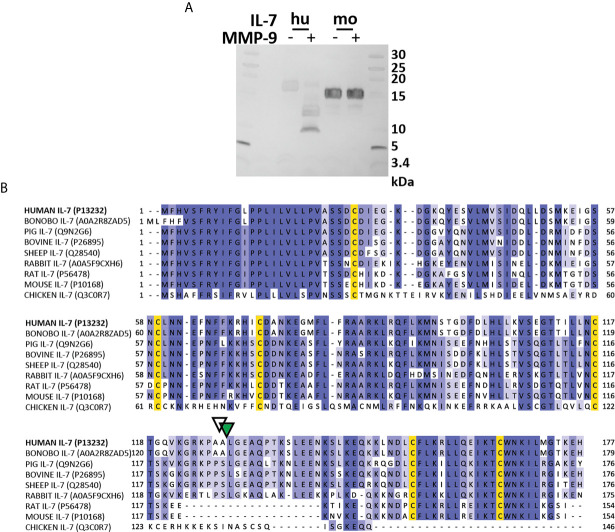
Mouse IL-7 lacks the protease-susceptible loop region and is not cleaved by MMP-9. **(A)** Incubation of recombinant human (hu, insect cell-derived) and (mo, *E. coli*-derived) IL-7 with active MMP-9 and analysis of the reaction products by reducing SDS-PAGE. **(B)** Alignment of the amino acid sequence of human, bonobo, pig, bovine, sheep, rabbit, rat, mouse and chicken IL-7 with indication of the MMP-9 (green triangle) and serine protease (white triangle) cleavage sites in human IL-7. Multiple sequence alignment was performed with ClustalO, with relative amino acid sequence conservations indicated in various shades of blue and conserved cysteine residues in yellow. Notice the missing sequences in mouse and rat IL-7 and a different sequence in chicken IL-7 in the area which represents the protease-sensitive loop.

Next, we wanted to further investigate IL-7 proteolysis *in vivo* [*e.g.* in MMP-9 knock-out (KO) mice]. Given the differences in the IL-7/IL-7R system function between humans (T-cell development) and mice (T-cell and B cell development) (32), we evaluated whether murine IL-7 was also susceptible to proteolysis by MMP-9. Mouse IL-7 was not cleaved by MMP-9 (**Figure 6A**). Interestingly, multiple sequence alignment of the IL-7 amino acid sequences of several species revealed that mouse and rat IL-7 lacked the protease sensitive loop region (**Figure 6B**). This finding points towards a difference in protease-sensitivity between murine and human IL-7.

## Discussion

Interleukin-7 is a critical regulatory T cell growth and survival factor in autoimmunity and cancer (9, 10, 32). Although the production and signaling cascade of IL-7 and its receptors are well established (9, 32), limited information exists about its elimination or half-life. We established that, within the human γ_c_/CD132 ligand family (9), IL-2, IL-7 and IL-21 are efficiently cleaved by MMP-9, whereas IL-4, IL-9 and IL-15 are not (21). In addition, IL-2 loses signaling functions after proteolysis by MMP-9 and by combined protease activities present in neutrophil degranulates, but for the other ligands this is not yet known (21). Here, we documented (i) the biochemical details of IL-7 processing by neutrophil enzymes; (ii) the biological effects of proteolysis on the binding affinities of IL-7 with its receptor alpha chain, on receptor-mediated signaling functions and on the growth of a T cell line as a biological read-out for IL-7 activity. By comparison of intact and cleaved IL-7 and of various IL-7 glycoforms, we were able to establish that (iii) N-linked oligosaccharides protected against proteolysis and that (iv) the function of disulfide bridges between cysteines was to keep the molecule in a signaling conformation after proteolysis in the human species, whereas (v) in other species, including mice and rats, the biology of these interactions may be completely different.

First, we determined that the loop region between the α-helices C and D was the most sensitive to proteolysis. Specifically, an efficient cleavage site for MMP-9 was identified between amino acid residues Ala^128^ and Leu^129^. At an MMP-9/IL-7 molar ratio of 1:100 almost complete cleavage of IL-7 was achieved within two hours, indicating that this *in vitro* cleavage was highly efficient in comparison with other known substrates of MMP-9, such as gelatins, actin and tubulin (36, 37). In addition, we identified a nearby cleavage site in IL-7 for NE and P3, situated between Ala^127^ and Ala^128^, exactly one amino acid N-terminally of the MMP-9 cleavage site in an accessible external loop. Interestingly, we also showed that the protease-susceptible loop region of IL-7 is not present in mouse and rat IL-7. This finding is particularly interesting given the known immunological differences between mice and men (38). In the context of our study, the most interesting differences relate to neutrophil abundancy and the IL-7/IL-7R system. In human blood neutrophil counts outnumber these in mouse blood (*vide infra*). As for the IL-7/IL-7R system, in humans, this system mainly controls T-cell development and in mice both T-cell and B cell development (32).

Cleavage of various glycoforms of IL-7 by MMP-9 resulted in the generation of two stable fragments, distinguishable upon reducing SDS-PAGE. We tested three different recombinant forms: aglycosyl IL-7 from *E coli*, IL-7 with insect cell glycans and IL-7 with the ensemble of glycoforms produced in a mammalian cell line. In accordance with such differences in glycosylation, the intact IL-7 as well as the cleaved fragments migrated at different molecular weights and were differently stained with the use of Coomassie brilliant blue after electrophoretic separation under reducing conditions. Furthermore, under non-reducing conditions, these fragments remained attached through the presence of internal disulfide bridges, keeping the IL-7 fragments interconnected. As a consequence, the overall structure remained intact and IL-7 cleaved by MMP-9 remained able to induce IL-7R internalization, to activate signal transduction through pSTAT3 and to induce cell proliferation in the human T cell leukemia cell line HPB-ALL. In addition, in primary human CD8^+^ PBMCs, stimulation with intact IL-7 or MMP-9-cleaved IL-7 resulted in a similar decrease of the IL-7R at the cell surface.

We showed that this phenomenon was not limited to MMP-9 and that stable fragments were also found after digestion of IL-7 with other MMPs (MMP-2 and MMP-8), ADAM17, NE and P3 and that, also under these conditions, cleaved IL-7 remained capable of inducing IL-7R-mediated cell proliferation. It is interesting to speculate what the function is of this IL-7 modification. One possible explanation is alteration of IL-7 half-life under specific circumstances such as altered redox state of the cellular environment or compartment. For example, cleaved IL-7 might lose structural integrity in reducing environments, resulting in separate fragments and altered IL-7 functionality. Such intracellular processing might considerably influence recycling of intact IL-7 *versus* cleaved IL-7. Additionally, reduction, extracellular modification of cysteine sulfhydryl groups (*e.g.* by oxidation with H_2_O_2,_ nitrosylation or sulfhydration), may lead to the generation of IL-7 fragments with altered function. Comprehensive follow-up studies covering these hypotheses and by further comparisons in cell culture systems with cell lines and specific primary cells including T-cell populations will yield further insights and can be relevant for both clinical targeting and parenteral use of recombinant cytokines, including IL-7.

Protein glycosylations, both by O-linked and N-linked oligosaccharides, are posttranslational modifications with the abilities to protect against proteolysis (39, 40). Originally described for the N-linked sugars of pancreatic ribonuclease being active in the protease-rich environment in the intestine (40), this function has been widened to many molecules in the internal milieu and even to intracellular glycoproteins. Oligosaccharides also co-determine the specific activities of enzymes and signaling molecules. This function was also described for glycosylated cytokines some 25 years ago (41). Although the half-life of signaling molecules, particularly those entering the blood circulation, is co-determined by glycosylation, this aspect received little attention so far for cytokines. Interestingly, it has been shown that glycosylation of the IL-7Rα ectodomain modulates the interaction of IL-7, with binding to glycosylated IL-7Rα, being 300-fold more tight than to unglycosylated IL-7Rα (29). Paradoxically, it was shown in another study that glycosylation of IL-7 does not affect its binding to or activation of the IL-7Rα (42). Here, we show that glycosylation of IL-7 reduces the efficiency of proteolysis by MMP-9. Aside regulation of IL-7 functions by expression levels, glycosylation as posttranslational modification adds an additional way of regulation to the IL-7/IL-7R system and its functions. Glycosylation studies of therapeutically used cytokines are gradually gaining attention. For instance, it is established that proteolysis of interferon-β (IFN-β) by MMP-9 is hindered by glycosylation (43) and that, maybe thereby, less neutralizing antibodies are formed in multiple sclerosis (MS) patients treated with glycosylated IFN-β (44). In line with this and the data in the present study, the glycosylation status of IL-7 needs careful attention, certainly, when IL-7 will enter therapeutic uses.

In a recent genome-wide association study (45), genetic alterations in the α-chains of IL-2 and IL-7 receptors (46, 47) were associated with an increased risk for MS. Hence, this information is suggestive for a key contribution of these signaling pathways to MS development and/or progression. In addition, based on expression patterns in lesions, sera and cerebrospinal fluid from patients with MS, the importance of proteases such as MMPs, NE, tissue kallikreins and cathepsins in MS has been established (48). Similarly, IL-7-mediated signaling contributes to leukemia development (32, 49) and cancer cells typically have increased production of proteases such as MMP-9 and other MMPs (50). Therefore, modulation of IL-7 by proteases secreted by infiltrating leukocytes or cancer cells are a plausible process both in MS and in cancer pathologies and these elements justify further investigation of the functional implications of IL-7 proteolytic processing. Interestingly, not many natural mutations in the *IL7* gene have been identified. A natural human variant of IL-7 has been described which lacks the C-terminal part of the IL-7 protein (introduction of a central stop codon R69X) and the corresponding homozygous genotype results in a disease called epidermodysplasia verruciformis-5 (EV5, Online Mendelian Inheritance of Man/OMIM 618309) (51).

One of the limitations of our study is the proof of MMP- and SP-generated fragments *in vivo*. However, the lack of sensitive and specific detection methods for differential quantification of cytokine and chemokine fragments *in vivo* is a well-known problem. For instance, with commonly used ELISA technologies one is not able to discriminate between intact and cleaved cytokines and Western blot analysis is less sensitive and only semi-quantitative. Aside this aspect, with the present information about structural differences between mouse and human IL-7, the common use of preclinical mouse animal models in immunology research comes with some pitfalls, both at the cellular and molecular level. At the level of inflammatory cells, in particular neutrophils being the prominent cell type in the present study, major differences exist between mice and humans. In human physiology and most pathologies, neutrophils represent the majority (50-70%) of circulating leukocytes and thus are the first and most abundant contributors in acute infections and inflammations (52). In the mouse species, neutrophil numbers are overshadowed by those of lymphocytes, thereby triggering a critical attitude towards these differences in translational research. At the level of interacting molecules, we provide here seminal information about differences across species with the aim to interpret *in vivo* data in an unbiased way and to remain critical in translation of phenotypes from one to another species.

Our work may also be placed in the context of recent clinical applications of IL-7 signal blockade for the treatment of leukemia, lymphoma and autoimmune diseases. Recently, it was found that endogenous IL-7 increases the expression of an oncogenic kinase (PIM1) in cells with the IL-7 R alpha chain (IL-7Rα/CD127). This was observed in both T-ALL and T-cell acute lymphoblastic lymphoma (T-LBL) (53). Secondly, the importance of IL-7 secretion by human primary T-ALL cells, as an autocrine positive feedback stimulus, was only recently evidenced and this finding incites further studies to interfere at the IL-7/IL-7R interfaces (54). The latter approach is reaching maturity in excellent new studies in which patient-derived xenotransplanted human T-ALL cells are killed with a chimeric monoclonal antibody against human IL-7Rα/CD127 *in vivo via* antibody-dependent cell-mediated cytotoxicity (55). Our findings have also consequences for autoimmune disease research, in which protease-cytokine interactions play multiple roles (56). In a recent study of alopecia areata, a T cell-mediated autoimmune disease of the hair follicles, IL-7 blockade was found to suppress acute inflammatory responses of the disease, while relatively sparing Treg (57).

In conclusion, we show that human IL-7 is efficiently processed by neutrophil enzymes within a protease-sensitive loop, generating disulfide-linked proteoforms retaining receptor binding and signaling functions. Human IL-7 is a glycosylated cytokine and its attached glycans protect it against proteolysis by neutrophil proteases. Posttranslational modifications, including proteolysis and glycosylation, deserve more attention in basic and applied research of cytokines. Finally, we show that mouse IL-7 does not contain the protease-sensitive loop and, consequently, is not cleaved by MMP-9. With this finding we further reinforce the known differences in IL-7 biology between the human and mouse species.

## Data Availability Statement

The datasets presented in this study can be found in online repositories. The names of the repository/repositories and accession number(s) can be found in the article/**Supplementary Material**.

## Author Contributions

Designed the study: GO, JV, VR, RVSP, and EU-B. Performed experiments and analyzed data: all authors. Wrote the manuscript: GO, JV, and VR, with input from all authors. All authors contributed to the article and approved the submitted version.

## Funding

This research was supported by the Research Foundation Flanders/FWO-Vlaanderen (G0A3820N), C1 funding of KU Leuven (C16/17/010), the Belgian Charcot Foundation (JV and GO), and the Rega Foundation (VR, JV). MM is supported by a predoctoral research fellowship “For Women in Science” of L’Oréal-UNESCO-FWO. There is no conflict of interest. JV is a postdoctoral fellow of the Research Foundation of Flanders (FWO Vlaanderen, mandate 12Z0920N).

## Conflict of Interest

The authors declare that the research was conducted in the absence of any commercial or financial relationships that could be construed as a potential conflict of interest.
