# Supplementary material for: Internal Disulfide Bonding and Glycosylation of Interleukin-7 Protect Against Proteolytic Inactivation by Neutrophil Metalloproteinases and Serine Proteases
Source: Front Immunol. 2021 Jun 30;12:701739. doi: 10.3389/fimmu.2021.701739 (PMC8278288; doi:10.3389/fimmu.2021.701739)
Supplement: Supplementary file 1 [file DataSheet_1.docx]

Supplementary Material

Internal disulfide bonding and glycosylation of interleukin-7 protect against proteolytic inactivation by neutrophil metalloproteinases and serine proteases.

Jennifer Vandooren^1^, Rafaela Vaz Sousa Pereira^1^, Estefania Ugarte-Berzal^1^, Vasily Rybakin^1^, Sam Noppen^2^, Melissa R. Stas^1^, Eline Bernaerts^1^, Eva Ganseman^3^, Mieke Metzemaekers^3^, Dominique Schols^2^, Paul Proost^3^, Ghislain Opdenakker^1*^

^1^Laboratory of Immunobiology, Rega Institute for Medical Research/KU Leuven, Department of Microbiology, Immunology and Transplantation, Leuven 3000, Belgium

^2^Laboratory of Virology and Chemotherapy, Rega Institute for Medical Research/KU Leuven, Department of Microbiology, Immunology and Transplantation, Leuven 3000, Belgium

^3^Laboratory of Molecular Immunology, Rega Institute for Medical Research/KU Leuven, Department of Microbiology, Immunology and Transplantation, Leuven 3000, Belgium

*Corresponding Author:

Ghislain Opdenakker
ghislain.opdenakker@kuleuven.be

**Supplementary Table 1:** Overview of human IL-7 sources, their expression systems, glycosylation levels and amino acid sequences

| **IL-7 source** | **Glycosylation** | **Supplier, cat. no.** | **Amino acid sequence** |
| --- | --- | --- | --- |
| **Uniprot database** | - | Entry, P13232 | **DCDIEGKDGKQYESVLMVSIDQLLDSMKEIGSNCLNNEFNFFKRHICDANKEGMFLFRAARKLRQFLKMNSTGDFDLHLLKVSEGTTILLNCTGQVKGRKPAALGEAQPTKSLEENKSLKEQKKLNDLCFLKRLLQEIKTCWNKILMGTKEH** |
| ***E. Coli*** | none | Peprotech, 200-07 | M**DCDIEGKDGKQYESVLMVSIDQLLDSMKEIGSNCLNNEFNFFKRHICDANKEGMFLFRAARKLRQFLKMNSTGDFDLHLLKVSEGTTILLNCTGQVKGRKPAALGEAQPTKSLEENKSLKEQKKLNDLCFLKRLLQEIKTCWNKILMGTKEH** |
| **insect cells** | partial | Novus, NBP2-52629 | ADP**DCDIEGKDGKQYESVLMVSIDQLLDSMKEIGSNCLNNEFNFFKRHICDANKEGMFLFRAARKLRQFLKMNSTGDFDLHLLKVSEGTTILLNCTGQVKGRKPAALGEQPTKSLEENKSLKEQKKLNDLCFLKRLLQEIKTCWNKILMGTKEH**HHHHHH |
| **HEK-293 cells** | full | Biolegend, 581904 | **DCDIEGKDGKQYESVLMVSIDQLLDSMKEIGSNCLNNEFNFFKRHICDANKEGMFLFRAARKLRQFLKMNSTGDFDLHLLKVSEGTTILLNCTGQVKGRKPAALGEAQPTKSLEENKSLKEQKKLNDLCFLKRLLQEIKTCWNKILMGTKEH**TGHHHHHHHHGGQ |

**
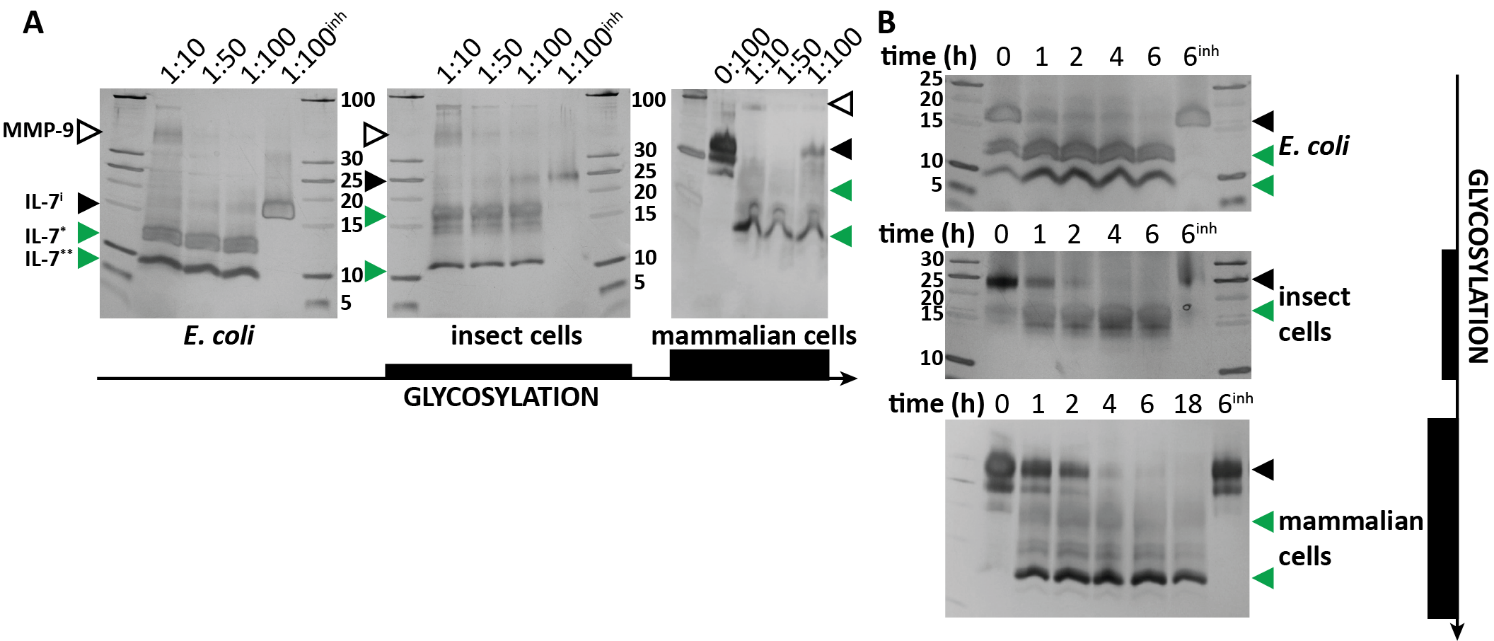
**

**Supplementary Figure 1: supplementary data on the digestion of IL-7 by MMP-9. A**, Repeat experiment of Figure 1A. Recombinant IL-7 produced in *E. coli* (left, *E. coli*), insect cells (middle, insect cell) or human HEK-293 cells (right, mammalian cells) with increasing complexities of N-linked sugars, were incubated with different concentrations of active MMP-9 at the indicated molar ratios (MMP-9/IL-7), for 2 hours. Fragments were resolved by SDS-PAGE under reducing conditions. MMP-9, intact IL-7 (IL-7^i^) and two cleavage products (IL-7* & IL-7**) are indicated with arrows. ^inh^; negative control with 500 µM of the MMP inhibitor SB-3CT. **B**, Repeat experiment of Figure 1B. Recombinant IL-7 produced in *E. coli* (top, non-glycosylated), insect cells (middle, simple insect glycoforms) or human 293E cells (bottom, complex mammalian glycoforms) were incubated with active MMP-9 at a molar ratio of 1:100 (MMP-9/IL-7) and reactions were terminated at the indicated time-points. Fragments were resolved by SDS-PAGE under reducing conditions. MMP-9, intact IL-7 (IL-7^i^) and two cleavage products (IL-7* & IL-7**) are indicated with arrowheads. ^inh^; negative control with 500 µM of the MMP inhibitor SB-3CT.

**
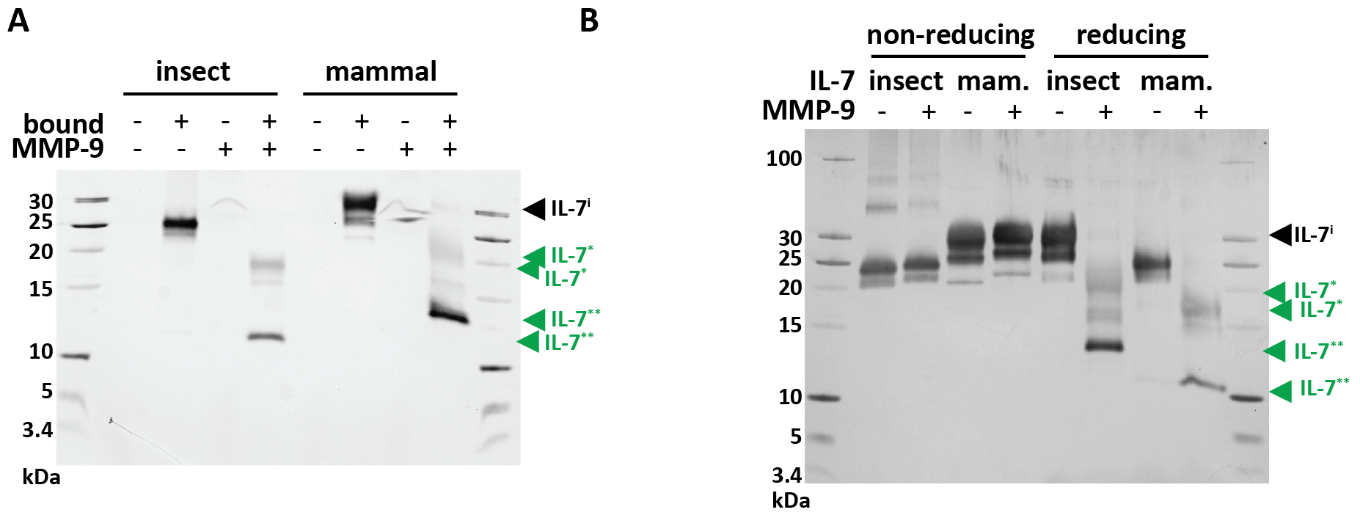
**

**Supplementary Figure 2: supplementary data on the characterization of MMP-9-generated fragments of IL-7. A**, Repeat experiment and full image of Figure 2A with differently glycosylated forms of human IL-7. His-tagged IL-7 (either insect cell or mammalian cell) was subjected to histidine-tag pull-down. Bead-bound material and non-bound material were resolved by reducing SDS-PAGE. **B**, Repeat experiment and full image of Figure 1B. IL-7 in the presence (+) or absence (-) of MMP-9, subjected to non-reducing or reducing electrophoretic separation. MMP-9, intact IL-7 (IL-7^i^) and two cleavage products (IL-7* & IL-7**) are indicated with arrowheads.

**
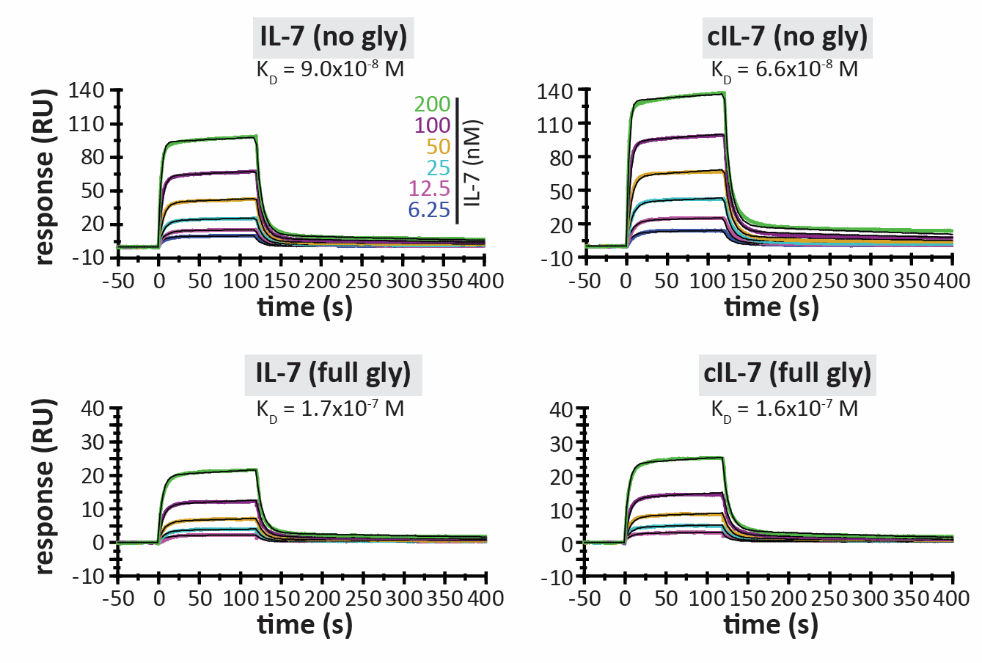
**

**Supplementary Figure 3: Binding kinetics of IL-7 and cIL-7 to IL-7Rα.** Repetition of results presented in **figure 2D** with another batch of MMP-9-digested IL-7. The various concentrations of IL-7 to generate the colored sensorgrams are indicated by their color keys and curve fittings are shown in black. Gly; glycosylation.

**
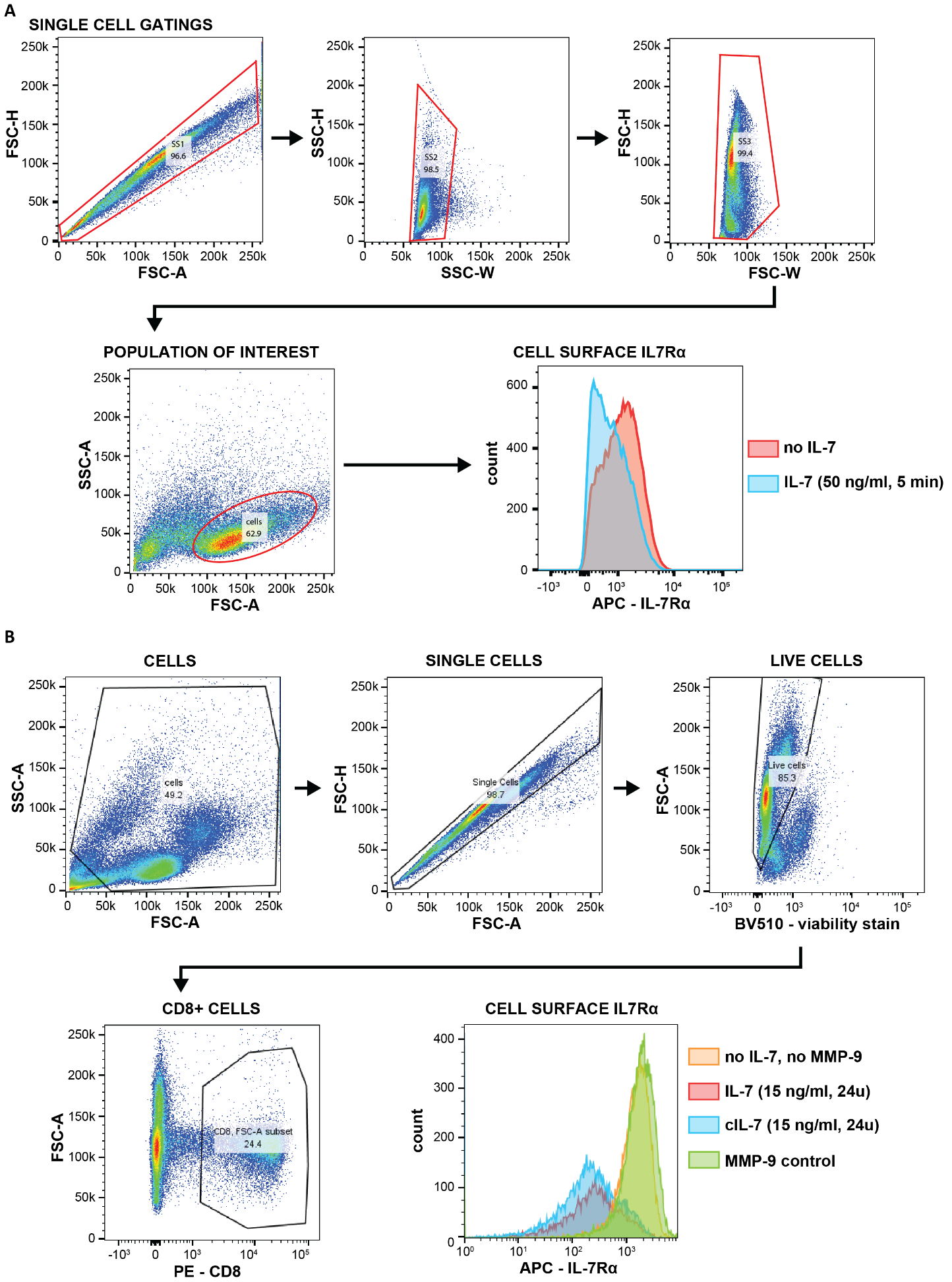
**

**Supplementary Figure 4: A,** Flow cytometry gating strategy for IL-7Rα staining in the HPB-ALL cell line. Representative flow cytometry dot plots and histograms. **B**, Flow cytometry gating strategy for IL-7Rα in human PBMCs. Representative experiments are shown.

**
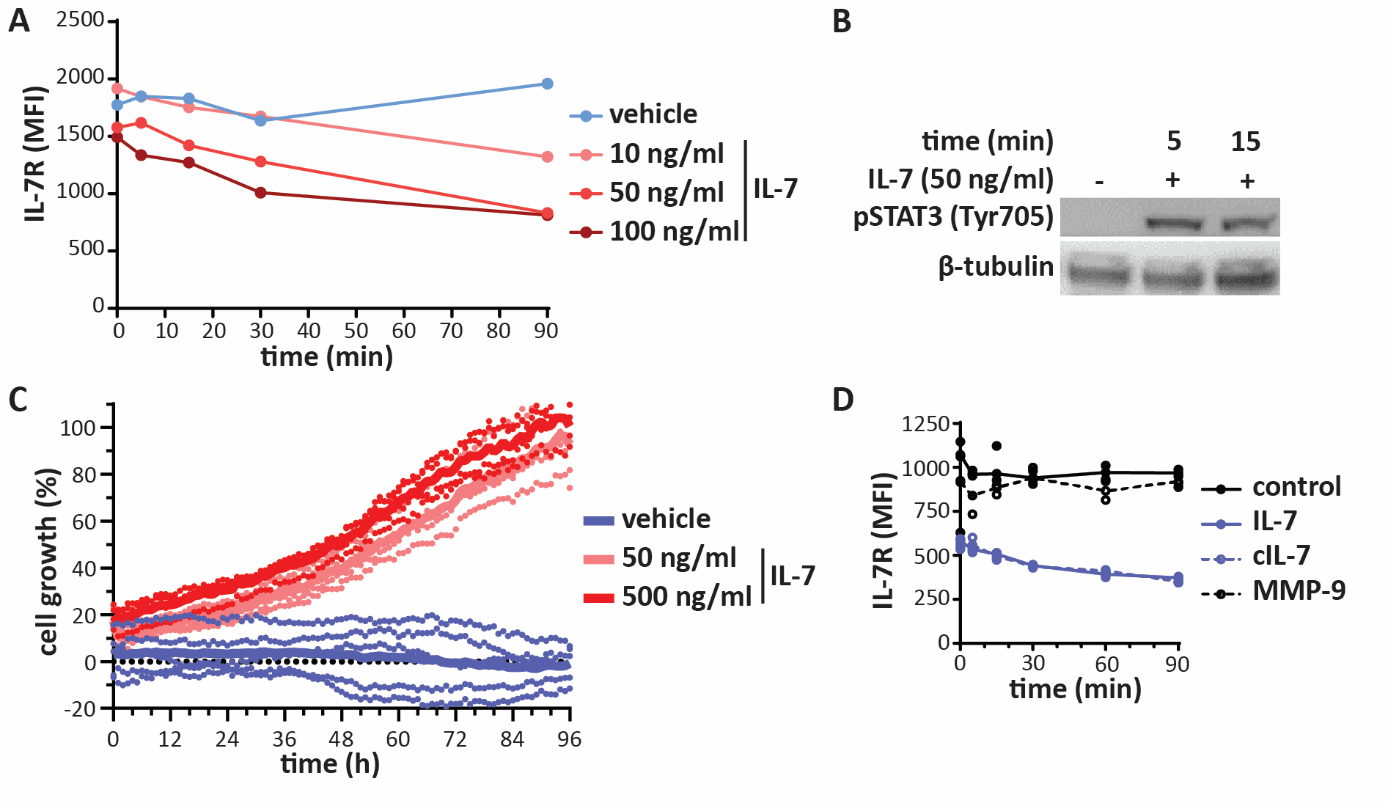
**

**Supplementary Figure 5: A,** Mean fluorescence intensity (MFI) of HPB-ALL cell-surface associated anti-IL-7Rα. Concentration- and time-dependent disappearance of IL-7Rα in response to human IL-7. Sample loading was controlled by re-probing the Western-blot for the presence of β-tubulin. **B**, Western-blot analysis of pSTAT (tyr705) and β-tubulin in HBP-ALL cells stimulated with IL-7. **C,** Growth curves of HPB-ALL cells in response to IL-7. Individual data points are shown and solid line indicates mean values (n = 5). **D,** Mean fluorescence intensity (MFI) of cell-surface associated anti-IL-7Rα. Time-dependent disappearance of IL-7Rα in response to human IL-7, cIL-7 (1/100, MMP-9/IL-7, 4h) or MMP-9 (n = 3).

**
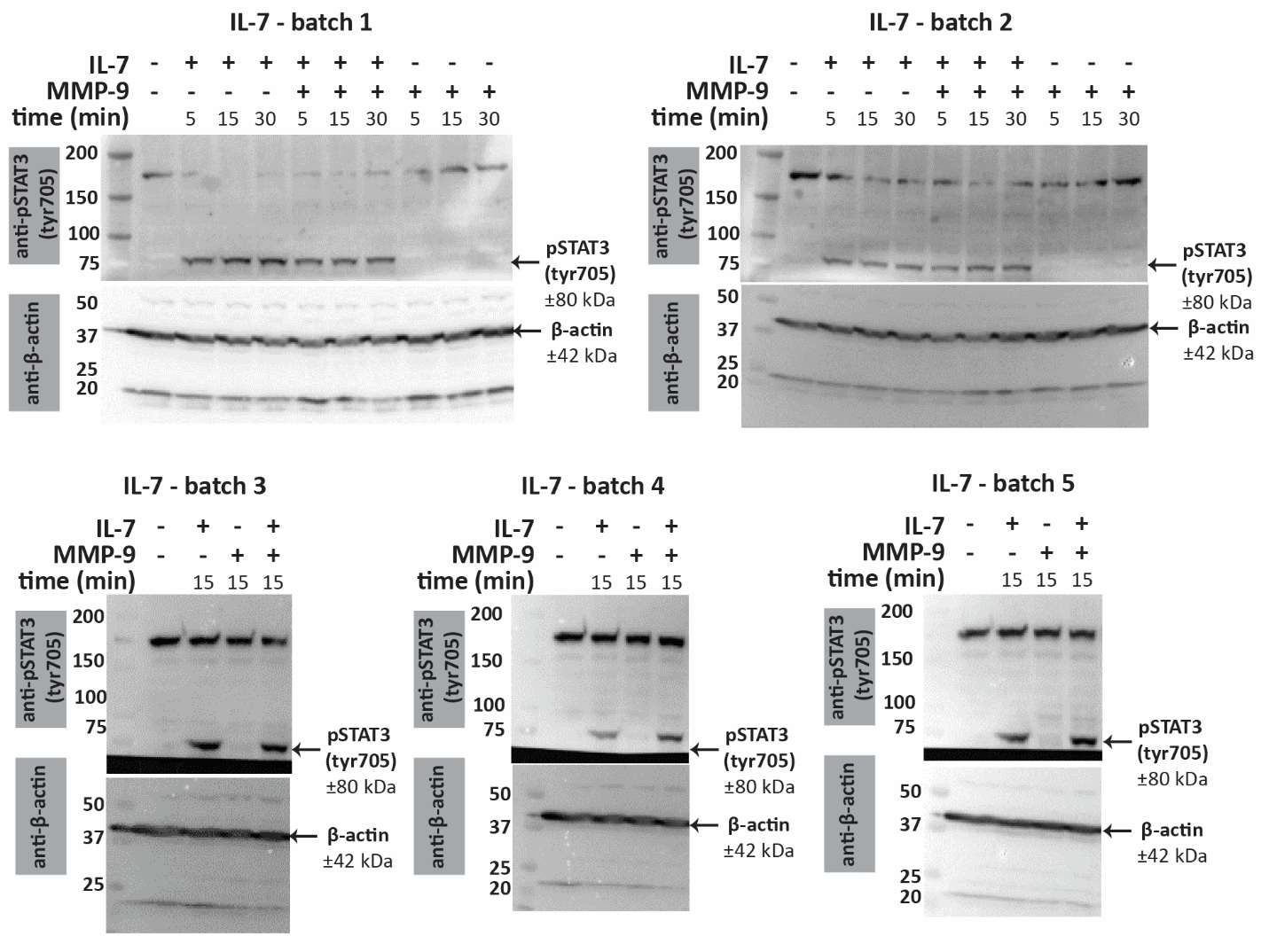
**

**Supplementary Figure 6:** full images and repeat experiments of the Western-blot analysis of pSTAT3 activation in response to IL-7 and MMP-9-cleaved IL-7 (Manuscript Figure 2F).

**
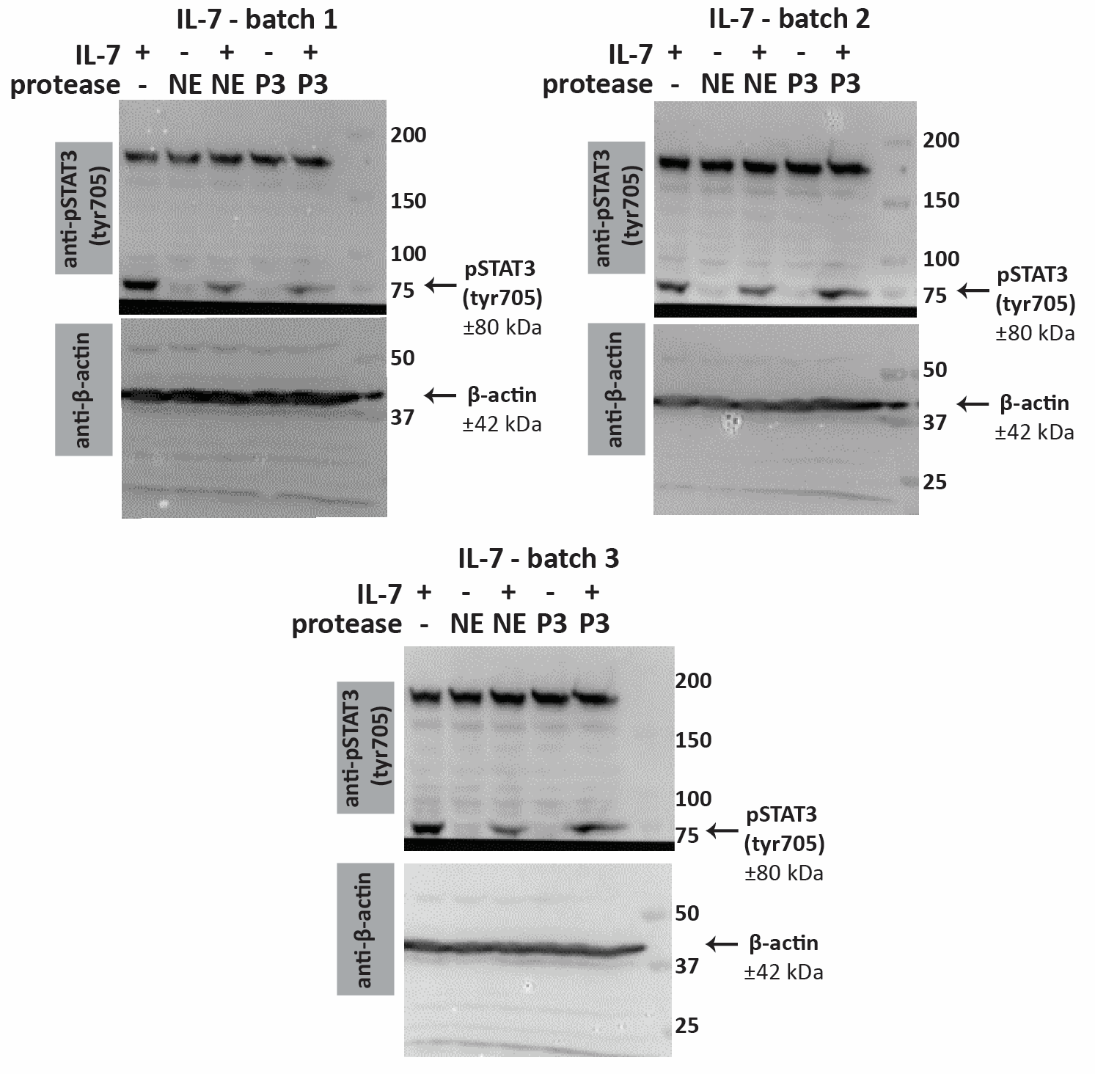
**

**Supplementary Figure 7:** full images and repeat experiments of the Western-blot analysis of pSTAT3 activation in response to IL-7 and neutrophil protease-cleaved IL-7 (Manuscript Figure 3H).
